# Mental ill health in structural pathways to women’s experiences of intimate partner violence

**DOI:** 10.1371/journal.pone.0175240

**Published:** 2017-04-06

**Authors:** Mercilene T. Machisa, Nicola Christofides, Rachel Jewkes

**Affiliations:** 1 Gender and Health Research Unit, South African Medical Research Council, Pretoria, South Africa; 2 School of Public Health, University of Witwatersrand, Johannesburg, South Africa; Massachusetts General Hospital, UNITED STATES

## Abstract

**Background:**

Depression, post-traumatic stress disorder (PTSD), and binge drinking are among mental health effects of child abuse and intimate partner violence (IPV) experiences among women. Emerging data show the potential mediating role of mental ill health in the relationship of child abuse and IPV. There is evidence that PTSD, depression and alcohol abuse are comorbid common mental disorders and that a bidirectional relationship exists between depression and IPV in some settings. Furthermore, the temporal direction in the relationship of alcohol abuse and women’s IPV experiences from different studies is unclear. We undertook a study with women from the general population to investigate the associations of child abuse, mental ill health and IPV; and describe the underlying pathways between them.

**Methods:**

Data is from a household survey employing a multi-stage random sampling approach with 511 women from Gauteng, South Africa. IPV was measured using the WHO Multi-country Study on Women's Health and Domestic Violence Questionnaire. Child abuse was measured using a short form of the Childhood Trauma Questionnaire. Depression was measured using the Centre for Epidemiologic Studies Depression Scale (CESD). PTSD symptoms were measured using the Harvard Trauma Questionnaire. Binge drinking was measured using the Alcohol Use Disorders Identification Test (AUDIT) scale. All data analyses were conducted in Stata 13. Regression modelling was used to test the association between variables. Structural equation modelling with full information maximum likelihood estimation accounting for missing data was done to analyse the underlying pathways between variables.

**Results:**

Fifty percent of women experienced IPV in their lifetime and 18% experienced IPV in the 12 months before the survey. Twenty three percent of women were depressed, 14% binge drank and 11.6% had PTSD symptoms. Eighty six percent of women had experienced some form of child abuse. Sociodemographic factors associated with recent IPV in multivariate models were younger age and foreign nationality. Depression, PTSD and binge drinking mediated the relationship of child abuse and recent IPV. Depression, PTSD and binge drinking were also effects of recent IPV. Other factors associated with recent IPV experience included relationship control, having a partner who regularly consumed alcohol and experiencing other life traumatic experiences

**Conclusion:**

Mental ill health plays a mediating role in the relationship of child abuse and recent IPV experiences among women. Conversely, IPV also negatively affects women’s mental health. Interventions to reducing the incidence of IPV could help alleviate the burden of mental ill health among women and vice versa. Effective integration of mental health services in primary health care, detection of symptoms, brief interventions and strengthened referral mechanisms for sustained community-based care are necessary in responding to victims of intimate partner violence. Response for abused children needs to take similar approaches and reduce the long-term mental health effects associated with violent exposures.

## Introduction

Violence against women (VAW) is a global public health problem and research has shown that 35% of women experienced physical or sexual intimate partner violence (IPV) or non-partner rape at least once in their lifetime [1]. Factors that increase risk for women’s victimisation include history of child abuse, witnessing intra-parent abuse, mental ill-health, unequal gender power relations and relationship conflict [2–7]. The social learning and intergenerational transmission theories for violence explain that IPV is higher among women who were abused in childhood or who witnessed parental violence[8]. The mental effects of child abuse can persist into adulthood and affect women’s overall health [9]. The long-term mental effects of child abuse include untreated depressive and post-traumatic stress disorder symptoms (PTSD) [9–12]. Child abuse is also associated with decreased self-esteem that increases risk for alcohol abuse and partner violence [11,13,14]. Depression, alcohol consumption, and PTSD increased women’s risk of experiences of IPV in some settings [1,15–17]. Women who have inequitable relationships and have less relationship power are at increased risk for IPV victimisation [18–21].

There is evidence that PTSD, depression and alcohol abuse are among the mental health effects of experiencing IPV [1,15,16,22–29]. Studies have shown a dose-response relationship in which PTSD and depression increased with the frequency and severity of IPV experiences [1,15,16,30–33]. Moreover, alcohol abuse is comorbid with PTSD, and depression in different contexts among women who have experienced IPV [32,33]. There is also evidence of strong associations between abusing alcohol, having a partner that abuses alcohol and women’s experiences of IPV [16]. Couple's drinking habits may pose as couple level risk factors for IPV [34,35]. In other instances mental ill health may affect women’s choices of partners with tendencies to select or stay with partners with greater risk of IPV perpetration [36].

The complex interrelationship of child abuse, mental ill health and IPV victimisation are apparent yet understudied and evidence is limited in the South African context [37]. Mental ill health is the third highest contributor to the burden of disease, after HIV and other infections disorders in South Africa [38]. Research conducted in South Africa has demonstrated the high prevalence, chronicity, and morbidity of mental ill health in a context where the health sector experiences challenges of integrating mental health services into primary health care [39–41]. Studies have demonstrated that South African children, adolescents and young people are exposed to high levels of violent trauma including child abuse, with a significant proportion developing PTSD, depression, suicidal notions and attempts [29,42–48]. Studies in South Africa have shown significant prevalence of emotional or psychological, economic, physical and sexual violence [27,49–52]. Lifetime prevalence of physical IPV reported by women from different studies ranged between 19% and 51% [7,51,53–55]. Physical IPV experiences by women were also associated with sexual IPV and the lifetime prevalence for sexual IPV was about 20% in different studies[55].

Mental ill health could play a mediating role in the relationship of child abuse and IPV victimisation in this context. Mental ill health could also be the effects of IPV experiences and moderate the risk for further re-victimisation. There is need to generate more evidence through investigating pathways between child abuse, mental ill health and IPV victimisation. This is important for identifying potential arena for violence prevention and response interventions.

We conducted a study to explore the potential mediating role of symptoms of mental ill health in the relationship between child abuse and women’s experience of IPV, in addition to describing the mental ill health effects of IPV victimisation. The study primarily aimed to determine the associations and underlying pathways between child abuse as a primary exposure and IPV experiences as an outcome and mental ill health measures as potential mediators of the relationship. The study a-priori hypothesis was that there are pathways from child abuse to IPV experiences mediated by PTSD, depression and alcohol abuse. We also hypothesised that the mental ill health measures would be comorbid and there would be associated with each other and with IPV experiences. We therefore assumed a possibility of bi-directionality between mental ill health and IPV implying that PTSD, depression and alcohol could increase women’s risk for IPV experience in addition to being effects of IPV experiences. By elucidating pathways between the interrelated variables, this study will be a significant addition to the body of existing literature [28,31,56–58].

## Methods

Researchers collected data through a household survey with a representative sample of women from Gauteng Province of South Africa in 2010. Gauteng province is the most populous and fastest growing of South Africa’s nine provinces with a population of 12.2 million people [59]. The province is mostly urban spanning the country's largest city, Johannesburg and its administrative capital, Pretoria, and surrounds.

The survey used a multi-stage random sampling approach to randomly select 40 enumeration areas (EA) using the South African 2001 National census as the sampling frame. Researchers randomly selected 20 households in each EA and invited only one adult woman residing in a selected household to participate in the study. Researchers reached 96% of the selected households and 89% of these households had an eligible participant. The response rate among women was 79%. Interviews were conducted with 511 eligible women who gave written consent to participate in the study[60].

The University of the Witwatersrand Faculty of Health Science Human Ethics Committee and the Medical Research Council Ethics Review Committee gave ethics approval for this study. All research was conducted conforming to safety guidelines for conducting research on domestic violence [61,62]. Interviews were conducted in private. Anonymous study identification numbers were allocated to participants and researchers assured them of confidentiality including that no data would be linked back to them. Researchers gave the women local referrals for support when they requested it [60].

The structured questionnaire was installed on personal digital assistants and administered by the researchers. Participants chose the language they preferred to respond in from English, Zulu, Sotho and Afrikaans.

### Measurement

#### Outcome variable

The main outcome of the study was physical, sexual, emotional or economic IPV in the past 12 months. We used the WHO Multi-country Study on Women's Health and Domestic Violence: Core Questionnaire and WHO Instrument–Version 9 to ask women about experiences of emotional abuse, physical violence and sexual violence [63]. We measured physical IPV by four items consisting of specific, violent acts perpetrated by a current or previous intimate partner with a weapon (**Table 1**). Three items of sexual violent acts by a current or previous partner (Table 1) measured sexual IPV. Six items that included different acts that were controlling, frightening, intimidating or undermined women’s self-esteem, measured emotional IPV. Acts of economic IPV included four items that included prohibition of engagement in income generating activities, having income taken and being evicted from a family home by a partner. Researchers asked participants about the frequency of these acts i.e. never (1), once (2), few (3) and many (4). We defined ever lifetime physical or sexual IPV experience as having experienced any of the physical IPV or sexual IPV acts once, few or many times. We measured IPV in the past 12 months also referred to here as recent IPV by a follow up question to the IPV subscale items. Participants who reported experiences of the acts of violence were asked, “Have any of these acts happened in the past 12 months?” and they had to respond with a “yes or no”. In this article, we define recent IPV as having experienced any acts of physical, sexual, emotional or economic violence in the 12 months before the survey.

**Table 1:** Variable and scale items.

Variable	Items
**Outcome**	
IPV in the past 12 months	Physical IPV items included slapping, throwing dangerous objects, pushing, kicking, hitting, dragging, choking, beating, burning or threatening. Sexual IPV items included physically forced non-consensual sex or sex because of fear of what a male partner might do or being forced to do something sexual by a male partner that they found degrading or humiliating. Emotional IPV included any of the following being insulted or made to feel bad about yourself, being belittled or humiliated in front of other people by a partner, a partner doing things to scare or intimidate on purpose for example by the way he looked, by yelling and smashing things, a partner threatening to hurt you or someone you love, a partner stopping you from seeing any of your family or friends and a partner boasting about or bringing girlfriends home girlfriends. Economic IPV included four items covering the following acts: being prohibited you from getting a job, going to work, trading, earning money or participating in income generation projects by a partner, a partner taking earnings from you, a partner forcing you or your children to leave the house where you were living and a partner not providing money to run the house or look after the children, but has money for other things.
**Explanatory**	
Child abuse	Fourteen items including: I did not have enough to eat; I lived in different households at different times; One or both parents were too drunk to take care of me; I spend time outside the home and none of the adults at home knew my whereabouts; Someone touched my buttocks or genitals or made me touch them when I did not want to; 1 had sex with a man who was more than 5 years older than me; 1 had sex with someone because I was threatened or frightened or forced and was forced to have sex against my will by a boyfriend; I was beaten at home with a belt or stick or whip or something else which was hard; I was beaten so hard at home that it left a mark or bruise; I was beaten or physically punished at school by a teacher; I saw or heard my mother being beaten by her husband or boyfriend; I was insulted or humiliated by someone in my family in front of other people; I was told I was lazy or stupid or weak by someone in my family.
**Mediating**	
PTSD symptoms	Thirty items including: recurrent thoughts or memories of most hurtful or terrifying events, feeling as though the event is happening again, recurrent nightmares, feeling detached or withdrawn from people, unable to feel emotions, feeling jumpy or easily startled, difficulties concentrating, trouble sleeping, feeling on guard, feeling irritable or having outbursts of anger, avoiding activities that remind of the traumatic or hurtful event(s), less interest in daily activities, feeling as if you have no future, avoiding thoughts or feelings associated with the traumatic or hurtful experience, sudden emotional or physical reaction when reminded of the most hurtful or traumatic event(s),feeling that people don’t understand what happened, difficulty performing work or daily tasks, blaming yourself for things that happened, feeling guilty for surviving, hopelessness, feeling ashamed of the hurtful or traumatic event (s), spending time thinking why these events happened to you, feeling as if going crazy, feeling that you are the only one who suffered, feeling others are hostile to you, feeling you have no one to rely on, being told by other people that you have done something that you cannot remember, feeling betrayed by someone you trusted and feeling split into two people with one watching what the other is doing.
Depression symptoms	Twenty items including: being bothered by things that usually don’t bother, not feeling like eating or poor appetite, unable to cheer up even with help of family or friends, feeling as good as other people, trouble keeping my mind on what I was doing, feeling depressed, feeling everything I did was an effort, feeling hopeful about the future, thinking my life was a failure, restless sleep, feeling happy, talking less than usual, feeling lonely, feeling that people were unfriendly to me, enjoying life, having crying spells, feeling sick, feeling that people dislike me and feeling unable to get going.
**Control**	
Sexual Relationship Power Score	Items include: If I asked my partner to use a condom, he would beat or hit me; If I asked my partner to use a condom, he would get angry; My partner won't let me wear certain things; My partner has more say than I do about important decisions that affect us; My partner tells me who I can spend time with; If I asked my partner to use a condom, he would think I'm having sex with other people; I could leave our relationship any time I wanted to; My partner does what he wants, even if I do not want him to; When my partner and I disagree, he gets his way most of the time; Because my partner buys me things he expects me to please him; My partner always wants to know where I am; My partner gets more out of our relationship than I do.
Other life trauma score	Ten lifetime events items including: imprisonment /detainment, civil unrest/war, serious injury requiring hospitalization, being close to death, witnessing a murder of family or friend, unnatural death of family or friend, witnessing the murder of stranger/s, torture, robbed or carjacked at gun or knife point and kidnapping. Responses to each trauma event were either “yes” or no.

#### Explanatory variable

Child abuse was measured using a 14 item modified version of the short form of the Childhood Trauma Questionnaire (CTQ) **(Cronbach’s alpha = 0.89)** [64,65]. Researchers asked participants whether they had experienced each act in their childhood (**Table 1**). Possible responses were “never” (1), “sometimes” (2), “often” (3), or “very often” (4). We summed up the responses for all 14 items of the CTQ to get a child abuse score. Answering at least “sometimes” to any of the items equivalent to a score greater than 14 was considered to mean the woman experienced a form of child abuse. **Table 1**shows the CTQ items and other scales items used in the study.

#### Mediating variables

Mental health variables included self-report measures of depression, PTSD, and binge drinking. Depression was measured using 20 items of the Centre for Epidemiologic Studies Depression Scale (CES-D) **(Cronbach’s alpha = 0.93**) **(Table 1)**. The CES-D self-report measure was designed for use in the general population to assess current symptoms of depression [66]. We asked women how often they experienced symptoms associated with depression over the past week and possible responses were “rarely or none of the time” (0) to “most or all of the times” (3). We summed up the item scores to create a continuous CES-D score that we use in all analyses. However, we dichotomized the score in order to describe the prevalence of depression in the studied population. As with previous South African studies we assumed that a score equal or greater than 21 indicated a high probability of clinical depression [65,67,68]

PTSD symptoms were measured using 30 items of the Harvard Trauma Questionnaire **(Cronbach’s alpha = 0.98**). The Harvard Trauma Questionnaire (HTQ) has been validated in other populations and cultural settings [69]. Researchers asked participants whether each of the symptoms had bothered them in the past weeks and could respond by “not at all” (1), “a little” (2), “quite often” (3) and “extremely often” (4) **(Table 1)**. We summed scores for all items to obtain a PTSD scale score. We calculated the PTSD DSMIV score by dividing the PTSD score with the number of items and assumed a mean score equal or greater than 2.5 to indicate significant PTSD symptoms as has been done with previous South African studies [30,57,67,70–72]

Binge drinking was measured using items from the Alcohol Use Disorders Identification Test (AUDIT) scale [73]. Researchers asked the participants whether they had drunk alcohol and their frequency of consuming five or more drinks on one occasion in the last year. Possible responses were never (1), less than monthly (2), monthly (3), weekly (4), and daily or almost daily (5). We considered a score of four or more (equivalent to drinking five or more drinks on one occasion weekly or daily) as binge or hazardous drinking.

#### Control variables

Socio-demographic data collected included age, education categorised into no schooling, primary, high school, tertiary; nationality and employment status in the year before the survey. We used the 11 item South African adaptation of the Sexual Relationship Power Scale (SRPS) **(Cronbach’s alpha = 0.92) (Table 1)** as a measure of gender imbalances [18,21]. We summed the responses for the SRPS items (Strongly Agree = 1, Agree = 2, Disagree = 3, Strongly Disagree = 4) to constitute a SRPS score. A lower SRPS score was indicative of more gender imbalance and less relationship power for the participant.

We measured other life trauma through an adapted 10 item Life Event Checklist from the PTSD Checklist (**Table 1**) [69]. We summed the scores for the items to create a continuous “other life trauma score” and controlled for this variable in analyses.

Another control factor measured was the alcohol consumption frequency of a current or most recent partner. Women were asked whether their current or most recent partner drank alcohol and how often this happened. Responses were never, a few times or less than once a month and every day or nearly every day. We recoded the responses to occasional drinking if a partner drank alcohol a few times or less than once a month and regular drinking if the partner drank at weekends, every day or nearly every day.

### Statistical analysis

We used Stata 13 for all analyses and took into account the stratified, two-stage survey design with participants clustered within the primary sampling units (PSUs). We excluded participants with missing data on key variables and recoded data to create derived variables that we used in analysis. The final study sample was 501 women aged 18 and above. We confirmed internal consistency for all the scales by examining the Cronbach’s alpha. We conducted bivariate analyses using cross-tabulations with measures of association adjusted for survey design to obtain the proportions of women experiencing IPV and mental ill health over the measured sociodemographic, child abuse and other life trauma variables. We present summary proportions disaggregated by both lifetime and recent IPV experience **(Table 2)**. We ran t-tests to compare the mean scores of explanatory variables among women that experienced vs those that did not experience IPV in the 12 months before the survey **(Table 2).** We ran mediation tests for mental ill health measures as potential mediators in the relationship of child abuse and recent IPV experiences using the seemingly unrelated regression (sureg) Stata command [74] **(Table 3).** We also used multivariable logistic regression modelling to test for factors associated with recent IPV and only included explanatory variables with p-values of less than 0.2 from the bivariate analyses. We used a backward elimination approach to remove non-significant variables until we obtained a parsimonious model **(Table 4)**.

**Table 2:** Bivariate analyses of socio-demographic characteristics, child abuse, other life trauma, mental ill health and women's experiences of IPV.

	No IPV in past 12 months n = 409			Any IPV in past 12 months n = 92			Total			P-Value
	%	95% CI		%	95% CI		%	95% CI		
**Age group**										
18–29	28.0	23.6	33.0	37.0	27.1	48.0	29.7	25.2	34.5	0.0018
30–44	34.6	29.0	40.7	46.7	36.4	57.4	36.9	32.5	41.4	
45+	37.4	31.0	44.2	16.3	10.7	24.1	33.5	28.0	39.5	
**Foreign National**	7.2	3.9	12.9	13.0	7.1	22.6	8.3	4.6	14.4	0.0054
South African	92.8	87.1	96.1	87.0	77.4	92.9	91.7	85.6	95.4	
**Education status**										
No schooling	5.4	3.7	8.0	1.1	0.2	7.1	4.6	3.2	6.7	0.0681
Primary school	18.8	14.3	24.4	10.9	5.4	20.7	17.3	13.1	22.7	
High school	62.6	57.3	67.7	73.9	63.9	81.9	64.7	59.8	69.4	
Tertiary	13.1	9.2	18.4	14.1	8.1	23.6	13.3	9.7	18.0	
**Employment and monthly earning past 12 months**										
Unemployed	55.7	49.0	62.2	58.7	45.8	70.5	56.3	50.5	61.8	0.7493
ZAR 0–2000	21.3	17.2	26.0	20.7	13.9	29.5	21.2	17.7	25.1	
ZAR2001-5000	11.7	8.6	15.6	13.0	6.9	23.4	11.9	8.9	15.7	
ZAR5001 +	11.4	7.2	17.5	7.6	3.4	16.2	10.7	7.0	16.0	
**Binge drinking**										
No	89.3	85.3	92.3	71.9	64.9	78.0	86.0	82.5	89.0	<0.0001
Yes	10.7	7.7	14.7	28.1	22.0	35.1	14.0	11.1	17.5	
**Partner alcohol**										
Never	52.6	48.0	57.1	31.5	23.6	40.7	48.7	44.9	52.5	0.0011
Occasional	16.6	12.8	21.4	19.6	12.6	29.2	17.2	13.7	21.3	
Regular	30.8	26.5	35.5	48.9	39.7	58.2	34.1	30.2	38.3	
**Child abuse score**	20.1	19.4	20.7	24.6	22.7	26.5	20.9	20.2	21.6	<0.0001
**Depression score**	14.3	13.3	15.2	22.6	20.2	25.0	15.8	14.9	16.8	<0.0001
**PTSD score**	22.9	21.9	23.8	31.9	29.5	34.3	24.6	23.7	25.5	<0.0001
**Other life trauma score**	1.0	0.9	1.2	1.7	1.3	2.1	1.2	1.0	1.3	0.0001
**Sexual Relationship Power Score**	32.3	31.5	33.0	26.4	24.6	28.3	31.0	30.3	31.8	<0.0001

**Table 3:** Mediation effects from seemingly unrelated regression test.

	Coefficient	95% CI		P-value
Child abuse→ Depression	0.39	0.26	0.51	<0.0001
Child abuse →PTSD	0.45	0.33	0.57	<0.0001
Depression → IPV past 12 months	0.01	0.001	0.01	0.022
PTSD → IPV past 12 months	0.01	0.003	0.01	0.002
Binge drinking→ IPV past 12 months	0.18	0.08	0.27	<0.0001
Child abuse → IPV past 12 months	0.01	0.00	0.01	0.001

**Table 4:** Multivariate regression models of factors associated with IPV in past 12 months.

	OR	95% CI		P-value
**Age group: 18–29**				
30–44	1.12	0.51	2.47	0.77
45+	0.26	0.09	0.75	0.013
**Nationality: Non-South African**				
South African	0.42	0.20	0.87	0.02
**Child abuse score**	1.01	0.96	1.06	0.661
**Other life trauma score**	1.22	1.01	1.46	0.04
**Binge drinking**	2.94	1.77	4.88	<0.0001
**CESD score**	1.04	1.01	1.06	0.004
**PTSD Score**	1.04	1.01	1.07	0.003
**Partner alcohol: Never**				
Occasional	1.62	0.67	3.89	0.272
Regular	1.57	0.93	2.64	0.088
**Sexual Relationship Power Score**	0.91	0.85	0.98	0.009

We specified a path model to analyse the underlying pathways that included the mediation of mental ill health based on the theoretically informed a priori hypothesis and the observed associations that were significant in the prior regression models. We also estimated a second path model that assumed mental ill health as being the effects of IPV. For both models we used SEM with full information maximum likelihood estimation accounting for missing variables[75]. The model building approach included first building measurement models for a child abuse latent variable allowing the indicators consisting of the CTQ scale items. We also built an “other-life trauma” latent variable consisting of eight of the items from the life-events checklist. For each latent variable, we conducted confirmatory factor analysis to optimise the indicators and allowed the indicators to co-vary until we obtained acceptable measurement model fit. We used the mental health variables as observed separately in the models. We conducted a sequence of model modifications including deleting non-significant paths until we obtained theoretically sound models with statistically significant pathways[75,76]. We allowed the errors of variables to co-vary in models when it was theoretically justifiable, this included the co-varying of errors of mental health variables. We controlled for the socio-demographic factors, nationality and age that were significantly associated with IPV in the regression models. We assessed the goodness of fit of the models by assessing comparative fit index (CFI> = 0.9) and Tucker-Lewis Index (TLI) (> = 0.9) and root mean square error of approximation (RMSEA< = 0.06) as indicative of acceptable fit [77,78].

## Results

The sample comprised 30% women aged 18–29 years, 36% were 30–44 years and 33% were over 45 years. The majority (91.8%) were South African nationals and the rest were foreign nationals. Sixty four percent had attended high school and only 44% had been employed in the 12 months before the survey. The majority (97.7%) had been in heterosexual sexual relationships and 78.7% were in a relationship at the time of the survey. Forty nine percent of women had current or most recent partners that did not take alcoholic drinks, 17% had partners who occasionally drank alcohol (once to a few times a month) and 34% had partners who consumed alcohol during weekends or daily.

Eighty six percent of women answered at least “sometimes” to one of the items on the CTQ and so had experienced a form of child abuse. Twenty three percent of women had depressive symptoms (CES-D score> = 21), 14.0% binge drank and 11.6% had PTSD symptoms (PTSD mean score > = 2.5). Thirty two percent of women had experienced one or more “other” traumatic event.

About one in five (18.4%) women had experienced any form of IPV in the 12 months before the survey and 13.8% of women had experienced physical or sexual IPV. Women experienced different forms of violence: 11.2% experienced all four forms of IPV i.e physical, sexual, economic and emotional; 2.6% of women experienced sexual, economic and emotional violence only; 2.4% experienced economic and emotional IPV and 2.2% experienced emotional IPV only in the 12 months before the survey.

**Table 2.** shows that higher proportions of younger women in the 18–29 and 30–44 year age groups and foreign nationals experienced IPV in the 12 months before the survey. Higher proportions of women who binge drank or who had partners that consumed alcohol occasionally or regularly reported recent IPV experiences. Women who experienced recent IPV also reported higher scores on the child abuse, PTSD, CESD and other life trauma scales. Women who experienced recent IPV reported lower scores on the Sexual Relationship Power Scale.

The tests for mediation effects showed that PTSD, depression and binge drinking gave statistically significant coefficients and confirmed that they were mediators in the relationship of child abuse and recent IPV **(Table 3).**

We tested for factors associated with IPV in past 12 months in a multivariate logistic model and included factors that had a p-value less than 0.2 in bivariate analyses. **Table 4.** shows that women who were 45 years and above were 74% less likely report recent IPV compared to women in the 18–29 age group. South African nationals were 58% less likely than foreign nationals to report IPV in the 12 months before the survey. There was no significant association between child abuse and recent IPV experiences. The risk for recent IPV increased with PTSD, depressive symptoms and other life trauma scores. The risk for recent IPV experiences was higher among women who binge drank. Recent IPV was not significantly associated with partner’s alcohol consumption. Higher scores on the sexual relationship power scale were protective from recent IPV experiences.

**Fig 1:**
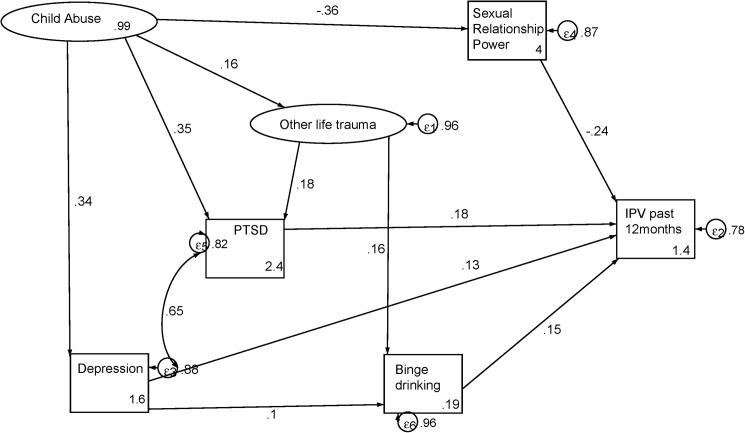
Mental ill health in pathways to IPV experiences.

**Fig 1.** shows that mental ill health mediated the relationship of child abuse and recent IPV. There were no direct pathways between child abuse and IPV, but there was one pathway mediated by depression and another mediated by PTSD. There was also a pathway mediated by both depression and binge drinking. Other life trauma events had indirect effects on IPV, mediating a pathway from child abuse through PTSD and another through binge drinking. Sexual Relationship Power also mediated the relationship of child abuse and recent IPV. The final modified model fit the data well (CFI = 0.92; TLI = 0.91; RMSEA = 0.04); p value = <0.0001). **Table 5.** shows the model statistics observed in the path diagram in **Fig 1**.

**Table 5:** Structural model statistics for mental ill health in pathways to IPV experiences.

	Standardized coefficients	SE	z	P>|z|	95% CI	
Child abuse→ Depression	0.34	0.04	7.87	<0.0001	0.26	0.42
Child abuse→ Sexual Relationship Power	-0.36	0.05	-7.98	<0.0001	-0.45	-0.27
Child abuse → Other trauma	0.16	0.07	2.28	0.022	0.02	0.29
Child abuse → PTSD	0.35	0.04	8.3	<0.0001	0.27	0.44
Other life trauma → PTSD	0.18	0.05	3.37	<0.0001	0.08	0.29
Depression → Binge drinking	0.10	0.05	2.24	0.03	0.01	0.19
Other life trauma→ Binge drinking	0.16	0.07	2.19	0.03	0.02	0.31
Depression → IPV past 12 months	0.13	0.06	2.24	0.03	0.02	0.23
Sexual Relationship Power → IPV past 12 months	-0.24	0.04	-5.66	<0.0001	-0.32	-0.15
PTSD → IPV past 12 months	0.18	0.06	3.15	<0.0001	0.07	0.29
Binge drinking → IPV past 12 months	0.15	0.04	3.79	<0.0001	0.07	0.23
**Disturbance variances**	**Estimate**	**SE**			**95% CI**	
Depression	0.90	0.03			0.84	0.95
Sexual relationship power	0.87	0.03			0.81	0.94
PTSD	0.84	0.03			0.77	0.91
Binge drinking	0.96	0.02			0.91	1.01
IPV past 12 months	0.78	0.03			0.72	0.85
Child abuse	0.99	0.01			0.97	1.01
Other life trauma	0.96	0.03			0.91	1.01
**Equation-level goodness of fit**	**r-squared**
Child abuse	0.008
Other life trauma	0.041
**Model goodness of fit statistics**	
p > chi2	<0.0001
RMSEA	0.040
CFI	0.922
TLI	0.910

**Fig 2:**
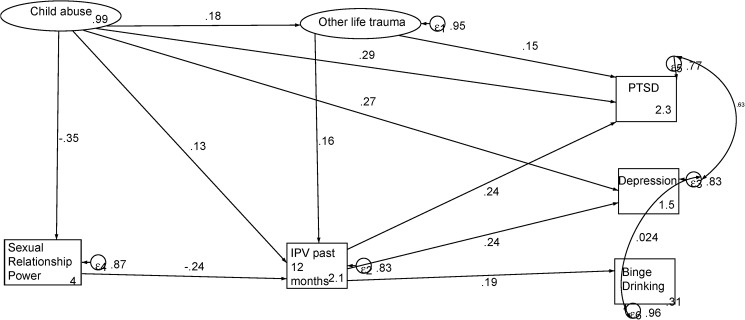
Mental ill health effects of IPV experiences.

**Fig 2.** shows the connections between child abuse and mental ill health. Child abuse had direct pathways to both PTSD and depression. There were also indirect effects of IPV experience and IPV mediated paths from child abuse, depression, PTSD and binge drinking. The model also shows the mediating effects of the sexual relationship power scale and exposure to other life traumatic events. In the model, the error of depression co-varied with those of both PTSD and binge drinking. **Table 6**shows the model statistics observed in the path diagram in **Fig 2**.

**Table 6:** Structural model statistics for mental ill health effects of IPV experiences.

	Standardized coefficients	SE	z	P>|z|	95% CI	
Child abuse →Depression	0.27	0.05	6.07	<0.0001	0.18	0.36
IPV past 12 months → Depression	0.24	0.04	5.83	<0.0001	0.16	0.33
Child abuse → Sexual Relationship Power	-0.35	0.05	-7.84	<0.0001	-0.44	-0.27
IPV past 12 months →PTSD	0.24	0.04	5.71	<0.0001	0.16	0.32
Child abuse→ PTSD	0.29	0.04	6.45	<0.0001	0.20	0.38
Other life trauma→ PTSD	0.15	0.05	2.81	0.005	0.04	0.25
Sexual Relationship Power→ IPV past 12 months	-0.24	0.05	-5.22	<0.0001	-0.33	-0.15
Child trauma→ IPV past 12 months	0.13	0.05	2.46	0.014	0.03	0.23
Other life trauma→ IPV past 12 months	0.16	0.06	2.60	0.009	0.04	0.28
IPV past 12 months → Binge drinking	0.19	0.04	4.43	<0.0001	0.11	0.28
Child abuse →Other trauma	0.18	0.07	2.64	0.008	0.05	0.32
**Disturbance variances**	**Estimate**	**SE**			**95% CI**	
Depression	0.86	0.03			0.80	0.91
Sexual Relationship Power	0.87	0.03			0.81	0.94
PTSD	0.80	0.03			0.73	0.87
IPV past 12 months	0.85	0.03			0.78	0.92
Binge drinking	0.94	0.03			0.89	0.99
Child abuse	0.99	0.01			0.97	1.01
Other life trauma	0.95	0.03			0.89	1.01
**Equation-level goodness of fit**	**r-squared**					
Child abuse	0.008					
Other life trauma	0.050					
**Model fit statistics**						
p > chi2	<0.0001					
RMSEA	0.04					
CFI	0.92					
TLI	0.91					

## Discussion

This study aimed to investigate the underlying pathways from child abuse to recent IPV. We hypothesised that comorbid mental ill health measures could mediate the relationship of child abuse and IPV in addition to being effects of IPV. In this study, we found that the significant inter-relationships of mental ill health measures mediated the relationship of child abuse and recent IPV. Child abuse had direct effects on PTSD symptoms and depression together with indirect effects on binge drinking. These findings show the persistence of mental ill health associated with childhood exposures of violence. Moreover, they conform to the theory of intergenerational transmission of violence. The findings also suggest bi-directionality in the relationship of IPV and mental ill health that is suggested in other studies[24]. PTSD, depression and binge drinking had direct effects on recent IPV and vice versa.

The study findings extend the existing literature by showing multiple pathways from child abuse to adult women’s experiences of IPV. The multiple pathways observed in this study reflect the potential to prevent women’s experiences of IPV by implementing different interventions and with different target groups. Of particular note is the role of mental health services and psychosocial support in addressing mental ill health among women. Interventions with women and girls that present with symptoms of mental ill health may have potential to reduce their further victimisation.

The prevalence of mental ill health among women substantiates the need for efforts to integrate mental health services into primary health care as a response to IPV in these settings. Nevertheless, other research in the same study settings has shown that health care providers do not take into consideration the patient’s illness narrative, miss the symptoms of mental ill health presented by victims of IPV, are uncertain of how to respond to IPV and lack knowledge of referral systems [79,80]. The result is that common mental disorders remain largely undetected and untreated in primary healthcare and this impact on the overall health of women [40]. Continued training of health care providers in targeted identification of symptoms of mental ill health and strengthened referral systems between health care service providers, psychosocial services and community based care are critical to curb the risk of women’s experience of IPV [81,82]. Collaboration between community based non-governmental organisations, stakeholders from the health sector, police, justice and community structures is necessary to strengthen a coordinated response and addressing child abuse, mental ill health and violence against women [40].

Other priority areas for the mental health and IPV response include the identification and reduction of barriers to women’s access to available services. This should include the promotion of mental health literacy to improve positive help-seeking behaviours for common mental health disorders [83]. Another potential entry point for addressing mental health could be in the provision of reproductive health services and Voluntary Counselling and Testing services. Women who are at a higher risk of IPV access these services and research has shown that women welcomed being screened for IPV in these settings [84]. Lay counsellor training could be tailored to include detecting mental ill health symptoms and provide the necessary referrals to mental health services providers [85].

These findings also show mental health as an effect of social problems. Addressing mental health in this context ought to go beyond a biomedical approach of treating symptoms to the development of brief culturally adapted interventions that can be administered in resource poor settings and help women to develop coping strategies or resilience [86]. The availability of effective referral systems to community-based care ensures sustainability of interventions. However, there is also need for adequate support, resourcing and capacity building for community-based organisations to be able to deliver quality psychosocial services.

The study findings showed a positive association between alcohol abuse and IPV that is consistent with previous literature. We also found that partner alcohol consumptions patterns were independently associated with recent IPV. Additionally our study showed the comorbidity of alcohol abuse with PTSD and depressive symptoms. In South Africa, alcohol abuse is also associated with structural factors that we have not accounted for in this study. Nonetheless, this study adds to the evidence suggesting that society-level strategies, couple level and individual level interventions that address harmful alcohol use are pertinent in reducing the prevalence of IPV in South Africa.

The study provides evidence of the need for primary interventions aimed to reduce child abuse or address its mental health effects among women and girls may have the potential to reduce lifetime and recent IPV. Secondary prevention may entail strengthening school and community based linkages to care for children with symptoms of common mental disorders or other mechanisms for mental health surveillance among children and youths [87,88]. Future research needs to investigate social support and personal characteristics that increase resilience to mental ill health and inform interventions for reducing women and girls’ risk to further victimisation [89,90].

This study also showed the significant contribution of other life trauma on PTSD, binge drinking and IPV experiences among women. Another study with men from the same population showed that experiencing of other life traumatic events exacerbated their perpetration of IPV and the relationship was also mediated by PTSD [91]. These findings reflect on South Africa’s societal context of high rates of interpersonal violence and crime impacting on violence in the home including child abuse and violence against women [92]. The findings are consistent with research showing that in communities with higher prevalence of community crime and violence, children and women are more vulnerable to abuse because social control is lower and violence has been normalised [93].

The mediation of sexual relationship power in the relationship of child abuse and IPV experiences is consistent with findings of previous studies showing associations between IPV and male controlling practices [19,31,94]. Scoring higher on the relationship power scale was protective of recent IPV risk. These findings highlight the continued need to address inequitable norms around gender relations in this context. However, given the findings from this study, interventions need to include both IPV prevention interventions focusing on empowering women and shifting in gender norms and the acceptability of violence, as well as and addressing mental ill health.

This study was limited in that it was cross-sectional and the models presented specifically focused on the relationship between child abuse, mental ill health symptoms and IPV experiences by women. The child abuse measure in the study is retrospective and may differ from the current incidence, however the findings reveal high prevalence of child abuse that could be averted by measures to mitigate corporal punishment of children and promote positive discipline at home and in schools[95]. The study did not include several other factors that have been fairly well established to be causally related to IPV at different levels of the integrated ecological model [96]. Therefore, these findings only relate to the inter-relationships of the observed variables and so do our conclusions and recommendations. While the data was from a survey and thus limited in establishing temporality, the use of the past 12 month estimated for IPV, current mental health allowed for testing of pathways from child abuse, a component that makes this study novel in our context.

Further research is necessary to investigate the observed relationships through longitudinal studies with larger samples, using clinical diagnoses and biomarkers for mental ill health and including other factors that are associated with abuse but not measured in this study for example poverty. Studies should employ analytical approaches to explore the multiple pathways and the overall impact of child abuse or mental ill health on IPV risk. Future psychosocial interventions should incorporate strategies to enhance women’s resilience to mental ill health in these settings and reduce women and girls’ risk of further victimisation. [89,90].

## Conclusion

This study has shown that mental ill health mediates the relationship of child abuse and IPV among women. Recent IPV is also associated with mental ill health effects. Treatment for mental disorders may form an important part of an effective package of interventions to prevent IPV, along with other gender transformative, parenting, and alcohol reduction programmes. IPV prevention programmes also have potential to reduce mental ill health among women. Evaluating current advocacy practice, developing and building consensus on effective, evidence-based violence reduction interventions needs to be a priority for improving the overall health of women in South Africa.
